# Supplementary material for: Investigating the Regulatory Mechanism of the Sesquiterpenol Nerolidol from a Plant on Juvenile Hormone-Related Genes in the Insect Spodoptera exigua
Source: Int J Mol Sci. 2023 Aug 28;24(17):13330. doi: 10.3390/ijms241713330 (PMC10488281; doi:10.3390/ijms241713330)
Supplement: Supplementary file 1 [file ijms-24-13330-s001.zip › ijms-2562426-supplementary.pdf]

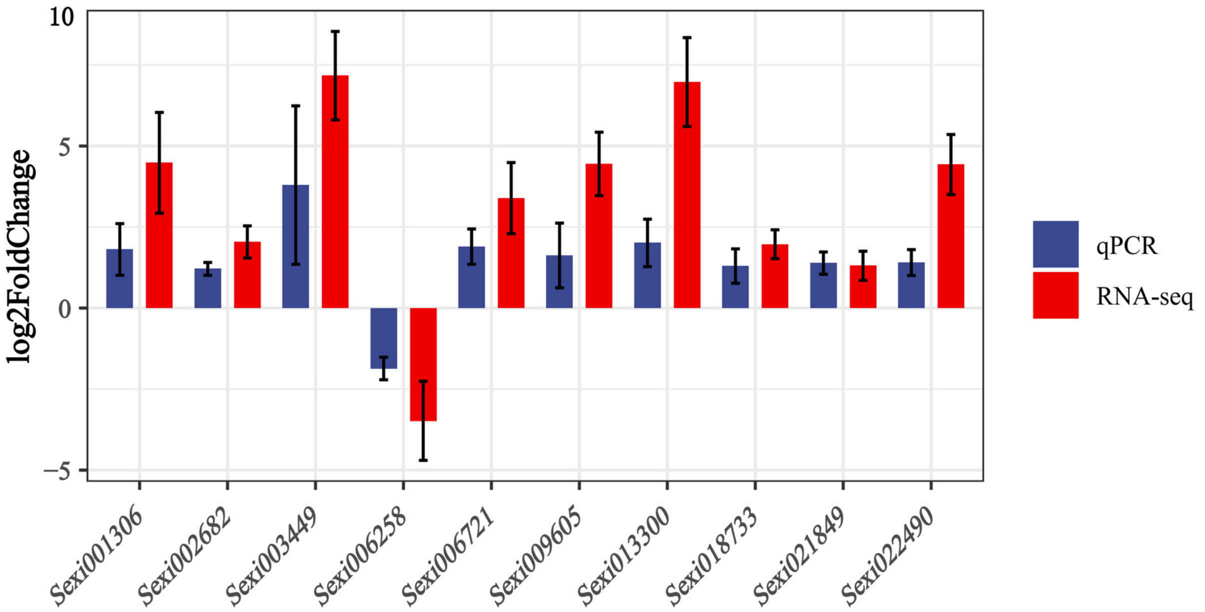

Figure S1. Verification of ten randomly selected differentially expressed gene using quantitative reverse transcriptase PCR.

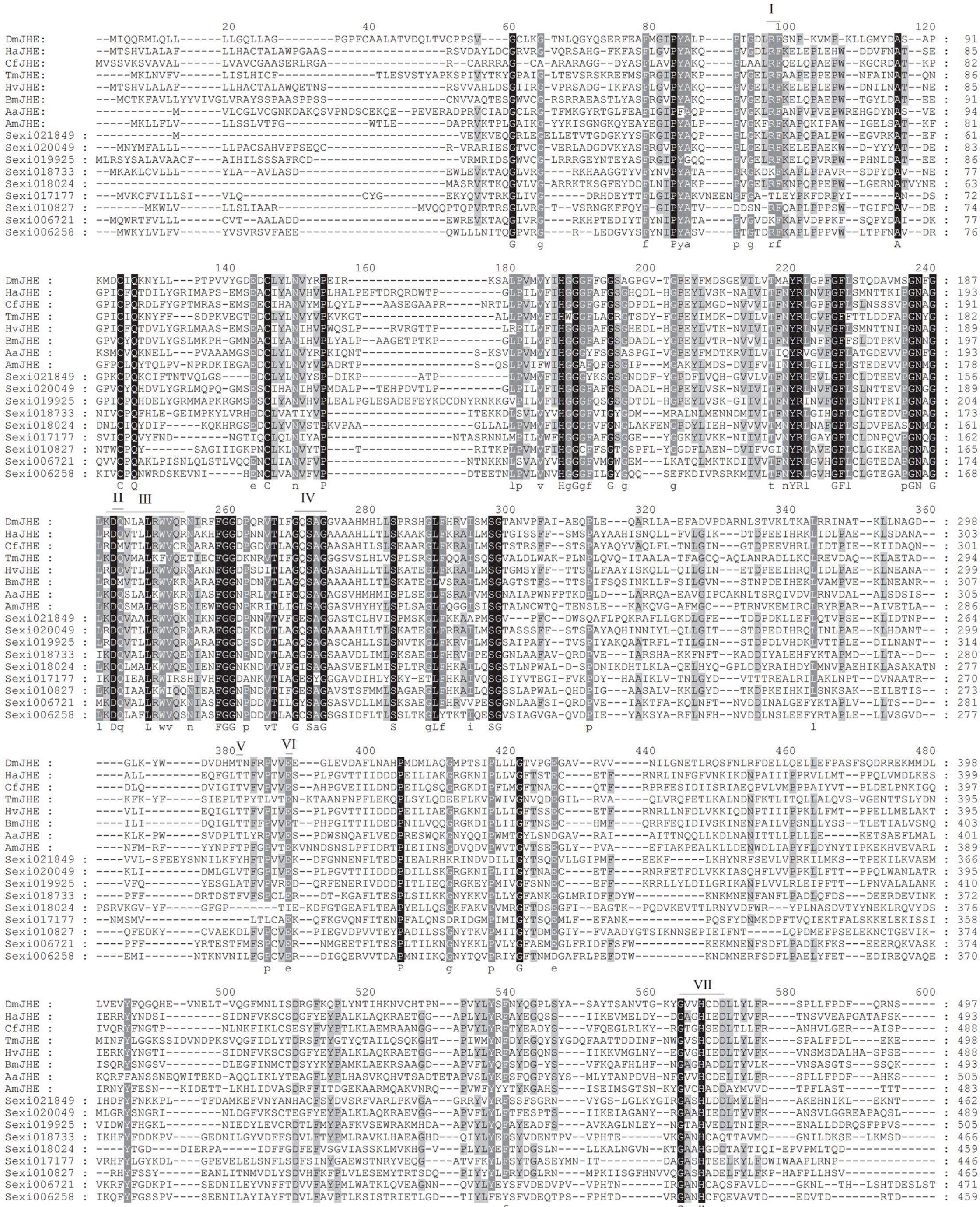

Figure S2. Nine JHE/JHEL candidate genes obtained from a genome screening of *S. exigua*. Multiple alignments (MAFFT version 7.143) of JHE sequences from various insect orders. The seven diagnostic motifs (I, II, III, IV, V, VI, and VII) and conserved amino acids for all JHEs are marked with gray solid lines.

Table S1. Conserved sequence motifs in the deduced *JHEs* and *JHELs* from various insect orders.

Source	Gene	SP [a]	Conservative Structural (Motif)							Others	
			RF	DQ	GQSAG	E	GxxHxxD	R/Kx(6)R/KxxxR	T	MW (kDa)	pI
Diptera	NP_001163166	p	+ [b]	+	+	+	+	Kx(6)RxxxR	+	64.52	6.30
	EAT43357	a	+	+	+	+	GxxHxxE	Kx(6)RxxxR	+	65.19	6.67
Coleoptera	AAL41023	p	+	+	+	+	+	Kx(6)KxxxE	+	65.89	5.24
Hymenoptera	NP_001011563	p	+	+	GLSAG	+	+	Kx(6)RxxxE	+	63.91	6.52
Lepidoptera	AEB77712	p	+	+	+	+	+	Rx(6)RxxxR	+	62.71	6.03
	AAD34172	p	+	DM	+	+	+	Rx(6)RxxxR	+	61.32	7.49
	AAC38822	p	+	+	+	+	+	Rx(6)RxxxR	+	62.57	5.36
	AAR37335	p	+	DM	+	+	+	Rx(6)RxxxR	+	62.72	6.38
S. exigua	*Sexi020049*	p	+	+	+	+	+	Rx(6)RxxxR	V	62.15	6.28
	Sexi019925	p	+	+	+	+	+	Rx(6)RxxxR	A	66.32	5.39
	Sexi021849	a	+	+	GESAG	+	+	Kx(6)RxxxK	Y	60.74	6.12
	Sexi018733	p	KF	+	GGSAG	+	GxxHxxQ	Kx(6)RxxxE	F	62.46	5.89
	Sexi018024	a	+	+	GISAG	I	+	Kx(6)KxxxE	No(gap)	59.66	6.00
	Sexi017177	p	TL	+	GESAG	+	GxxHxxE	Kx(6)RxxxS	No(gap)	59.93	7.17
	Sexi010827	p	+	+	GESAG	+	GxxHxxE	Kx(6)KxxxQ	D	63.09	7.19
	Sexi006721	p	KF	+	GYSAG	+	GxxHxxQ	Kx(6)RxxxK	F	63.26	5.91
	Sexi006258	p	+	+	GCSAG	+	GxxHxxQ	Kx(6)RxxxS	I	61.19	4.53

[a] : 'SP' represents 'signal peptide'. 'p' and 'a' represent the 'presence' and 'absence' of the signal peptide, respectively. [b]: The "+" indicates that the consensus sequence is maintained. NP_001163166.1 is the accession number of *DmJHE* (*Drosophila melanogaster*), EAT43357.2 of *AaJHE* (*Aedes aegypti*), AAL41023.1 of *TmJHE* (*Tenebrio molitor*), NP_001011563.1 of *AmJHE* (*Apis mellifera*), AEB77712.1 of *HaJHE* (*Helicoverpa armigera*), AAD34172.1 of *CfJHE* (Choristoneura fumiferana), AAC38822.1 of *HvJHE* (Heliothis virescens), and AAR37335.1 of *BmJHE* (*Bombyx mori).*

Table S2. Primers used for RT-qPCR.

Primer Name	Primer Sequence (5′-3′)	Lenghth of Product (bp)
β-actin—F	CCAGCCTTCCTTCTTGGGTAT	94
β-actin—R	AGGTCCTTACGGATGTCAACG	
Se021849—F	ATTCGGCCCAAAATGTCCAC	173
Se021849—R	AATCGTCGTTACCAGAGCCA	
Se018733—F	TGAAGTTACATGCGGAAGCTGGAC	81
Se018733—R	GCACCACAGGAGTATTCTCGTCAAC	
Se006721—F	TGTTACTGTTGTGCGTGACG	184
Se006721 -R	AATTTTGGTGGGTCAACGGG	
Se006258—F	GTTCAGTGTTCGCCGAAGAG	186
Se006258—R	GATCGACTGCATTGAACGGG	
Se002682—F	CATACGCCCGAG TGGATGCTTC	100
Se002682—R	CATCTGATTGTGCCGTCGTTTGC	
Se013300—F	TGAGTTCCGGCAAAACAGAC	181
Se013300—R	GCCATGTCTTCCCTGATCCT	
Se022490—F	TCTGACACCTACGCCAAAGA	181
Se022490—R	CCACAATGCGATCCAACCAT	
Se003449—F	CCGAAGACAACTGGAGCCAAGAC	150
Se003449—R	GTTCAGGAACTGTCGCTGGTGTAC	
Se001306—F	TTTGCGTGTCTTGTGTTGTGTGTTG	144
Se001306—R	AGCCTGGATCTGAGCGTCTGTG	
Se009605—F	CGAGAAGCAGAAGGGCGTGAAG	93
Se009605—R	TGGTCGAGAGTGTCCTTAGCAGTC	
